# Do Mothers Benefit from a Child-Focused Cognitive Behavioral Treatment (CBT) for Childhood Functional Abdominal Pain? A Randomized Controlled Pilot Trial

**DOI:** 10.3390/children4020013

**Published:** 2017-02-15

**Authors:** Claudia Calvano, Martina Groß, Petra Warschburger

**Affiliations:** 1 Counseling Psychology, University of Potsdam, Potsdam 14469, Germany; calvano@uni-potsdam.de; 2 Deutsche Morbus Crohn/Colitis ulcerosa Vereinigung (DCCV e.V.), Berlin 10179, Germany; mgross@dccv.de

**Keywords:** functional abdominal pain, parents, CBT, behavior, self-efficacy, RCT, waitlist control

## Abstract

While the efficacy of cognitive-behavioral treatment (CBT) approaches for childhood functional abdominal pain (FAP) is well-established for child outcomes, only a few studies have reported on parent-specific outcomes. This randomized controlled pilot trial analyzed effects of a group CBT on maternal variables (i.e., pain-related behavior, worries and self-efficacy, as well as general psychosocial strain). *Methods*: The sample constituted of 15 mothers in the intervention group (IG) and 14 mothers in the waitlist control group (WLC). Outcome measures were assessed pre-treatment, post-treatment and at three months follow-up. *Results*: Analyses revealed significant, large changes in maladaptive maternal reactions related to the child’s abdominal pain in the IG compared to the WLC—i.e., reduced attention (*d* = 0.95), medical help-seeking (*d* = 0.92), worries (*d* = 1.03), as well as a significant increase in behaviors that encourage the child’s self-management (*d* = 1.03). In addition, maternal self-efficacy in dealing with a child’s pain significantly increased in the IG as well (*d* = 0.92). Treatment effects emerged post-treatment and could be maintained until three months follow-up. There were no effects on general self-efficacy and maternal quality of life. *Conclusion*: While these results are promising, and underline the efficacy of the CBT approach for both the child and mothers, further studies, including long-term follow-ups, are warranted.

## 1. Introduction

Functional abdominal pain (FAP) is very prevalent in childhood [1], highly impairing [2] and persistent when untreated [3]. Not only do the children suffer from increased psychological distress [4,5] and a poor health-related quality of life [6,7], childhood FAP also affects the family functioning and is commonly associated with parental stress [8]. Furthermore, literature suggests that parents often influence a child’s coping and course of abdominal pain [8,9] therefore a parent`s reaction when the child experiences pain can be pivotal. This is especially true for parental protective behaviors, such as excusing the child from school or household chores and increased attention when their child is in pain—which are often seen as maladaptive behaviors—and can be associated with a child’s impairment [10,11,12] and increased healthcare use due to abdominal pain [13].

Current literature reports that a significant number of parents experience high psychological strain and a poor quality of life [14,15,16]. Taking this into account, analyses of treatment effects on parents’ psychosocial well-being should be of interest to researchers as well. The efficacy of cognitive behavioral treatment (CBT) approaches for FAP, in reducing a child’s pain and disability, is well-established [17,18,19,20]. Despite the critical role parents play in pediatric pain, comparatively few data exist focusing on parent-specific outcomes in relation to these approaches [21,22]. Results from controlled trials have suggested that significant effects on parental protective behavior and threat beliefs can be seen up to 12 months post-intervention [23,24,25]. A short-term improvement of maladaptive parental responses was also reported [25]. However, thus far, there is no data analyzing the effects on parents’ self-efficacy, which can be an important mediating mechanism underlying behavioral changes [26,27]. In pediatric pain research, self-efficacy refers to the perceived competence in dealing with pain in everyday life [28]. Analyzing this construct may extend current knowledge on parent-specific treatment effects [22] and could identify possible mediators for long-term sustenance of change.

This report aims to fill these gaps by analyzing the effects of an outpatient, manualized CBT group for children suffering from FAP (described in [29]), on the mothers’ behavior, worries, self-efficacy and quality of life. Data are derived from a randomized controlled trial (RCT) that included a waitlist control group (WLC) and three months follow-up. Positive, large-sized effects on self-reported pain, coping and health-related quality of life were observed in the children`s outcomes [30,31]. In line with these favorable effects on the affected children, we hypothesize that the mothers in the intervention group (IG) compared to the WLC, will report a more pronounced reduction of child’s pain and gastrointestinal (GI) symptoms.

Additionally, we hypothesize that mothers in the IG compared to the WLC will show a more pronounced reduction in: (1) attention, protective behavior, medical help-seeking; in (2) pain-related worries; and a more pronounced increase in (3) behavior encouraging child’s self-management; and in (4) their self-efficacy in dealing with the child’s pain. In an exploratory manner, we will analyze whether positive changes will also be observed in the mothers’ general self-efficacy and quality of life.

## 2. Materials and Methods

### 2.1. Study Design and Procedure

**Figure 1:**
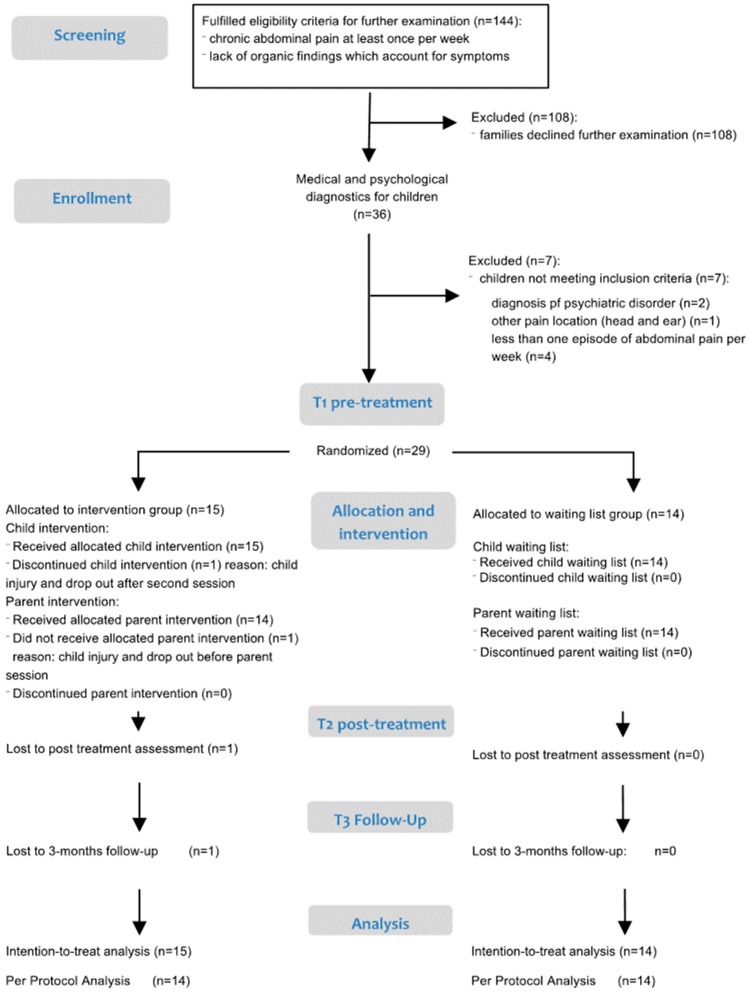
Trial flow chart. Adapted and extended for parent analysis from [31].

The study is a prospective RCT with a parallel waitlist design for the evaluation of a group cognitive behavioral program for children aged 6–12 years suffering from FAP (Current Controlled Trials registration ISRCTN 69830258). Figure 1 displays the RCT flow chart, which follows CONSORT guidelines. The complete CONSORT checklist is provided (Table S1) as an online supplementary document. Eligible families were recruited from an epidemiological study on the psychological well-being of primary school children over a 15-month period (recruitment: April 2008–July 2009). Eligibility criteria for further medical and psychological examination referred to the children, and were defined as the occurrence of chronic abdominal pain at least once a week and lack of organic findings which could account for the symptoms. The 144 eligible cases were invited for further examination to confirm self-reported questionnaire data. This included referring the child for a medical examination for the diagnosis of pediatric FAP and a psychological examination by a standardized diagnostic parent interview [32] to eliminate the possibility that the child fulfills criteria for a psychiatric disorder, according to ICD-10. For pain symptomatology, Rome-III diagnosis of Functional Abdominal Pain Syndrome (FAPS) [33] had to be fulfilled. Diagnosis of FAPS, according to Rome III, requires abdominal pain at least once per week for at least two months, along with associated impairment in everyday life or occurrence of other bodily complaints. A detailed description of the study design can be found in previous publications by the authors [30,31].

In total, 29 families were included and randomized to either the IG (*n* = 15) or the WLC (*n* = 14), applying a 1:1 allocation ratio. A computer-assisted randomization sequence was generated, with no restriction, and conducted by a person not involved in the study, and one of the authors enrolled the participants and assigned them to interventions based on the randomization results. Due to the waitlist control design, blinding was not possible. Data were collected at diagnostic examination (T0), two weeks pre-treatment (T1), 2 weeks post-treatment (T2) and 3 months post-treatment (T3). Data collection, as well as the intervention, took place in the outpatient Patient Training Center (PTC) for chronically ill children and their parents, associated with the Counseling Psychology Department of the University of Potsdam, over a 20-month period (interventions: April 2008–December 2009). In the IG, four groups were conducted consecutively. One child in the IG dropped out after the second child session due to an injury (broken leg) and the family did not participate any longer in the training program (the parents did not take part in the parent session, which was scheduled after the third child session) [29]. The participants in the WLC were offered a participation in the training program, only after completion of T3 assessments, which was approximately five months after inclusion. The study was approved by the Ethics Committee of the University of Potsdam.

### 2.2. Intervention

Families assigned to the IG received the manualized, cognitive-behavioral, child-centered group intervention entitled “Stop the pain with Happy Pingu” [29], including six group sessions for the children (weekly; each 90 min) and one parent group session (90 min). The primary aim of the training was to enhance the children’s self-management skills to cope with their pain. In order to attain this goal, children participated alone in the sessions without their parents, learned different skills to cope with their pain and were encouraged to do their regular homework (relaxation and practice of coping skills) on their own. In particular, children were instructed to apply cognitive-behavioral techniques, such as progressive muscle relaxation, distraction, cognitive restructuring and stopping negative thoughts [29,31].

The main goal of the parent session was to foster trust in the new competencies that the child had acquired. Parents received detailed information about the contents of the preceding sessions with the children and the biopsychosocial disease model underlying the intervention. Thereby, parents were asked to reflect on their own role in maintaining the disorder. The group training allowed the mutual exchange of personal experiences and the discussion of potential maladaptive reactions. In particular, parents were encouraged to rely on the competencies the child had learned and to support their child to implement these skills in their pain-coping activities. This was based on a mutual exchange led by the trainer, not on practices of behavioral responses. In addition, parents were informed how to recognize clinical alarm signals, according to the recommendations of the Subcommittee on Chronic Abdominal Pain in Children [34]. Since carbohydrate malabsorptions are common concerns in children suffering from FAP, nutritional guidelines were discussed, with the focus on a balanced diet [29]. This part of the parent session was conducted by a nutrition scientist, with the remaining contents, as well as the child sessions, conducted by one of the authors.

### 2.3. Measures

For this study, primary outcomes refer to pain-related measures (i.e., parental perception of pain and GI symptoms, parent’s behavior, worries and self-efficacy). Secondary outcomes refer to general self-efficacy and the parent’s quality of life. In addition, treatment satisfaction in the IG was also assessed. Except for the demographic and socioeconomic measures (T0), primary and secondary outcome data were assessed pre-treatment (T1), post-treatment (T2) and at 3 months follow-up (T3). Parents of the intervention group reported on treatment satisfaction for the intervention at post-treatment (T2).

#### 2.3.1. Demographic and Socioeconomic Measures

Child age, gender and number of siblings were reported by the mothers. In addition, child psychological distress was assessed in the parent report of the validated German version of the Strengths and Difficulties Questionnaire (SDQ) [35]. To evaluate its clinical impact, the total problem score was compared with German normative values; scores were classified as normal, borderline and abnormal psychological distress. Socioeconomic status (SES) was assessed according to the procedure defined by Blossfeld [36], which was a validated and broadly used measure to include the parent`s educational level and current work situation.

#### 2.3.2. Treatment Satisfaction

To assess treatment satisfaction, we adapted the parent version of the Therapy Evaluation Questionnaire [37], a validated questionnaire originally developed for the psychotherapy setting. In our study, 15 of the 21 items were adapted to properly fit to our context (i.e., “therapy”/”therapist” was replaced by “training”/”trainer” and “problem” was replaced by “pain”). The 21 items were split into two scales: treatment success (7 items, e.g., “I got a better understanding of child’s pain”; “I was able to positively change my behavior in the course of training”) and treatment process (14 items, e.g., “The trainer understood our situation”; ”I was able to openly talk to the trainer about the problems which made us come here”). Answer format was on a 5-point scale: (1) not at all/never; (2) hardly/seldom; (3) partly/sometimes; (4) predominantly/mostly; (5) exactly/always. According to the manual, we also calculated the total score as the mean of all items. The questionnaire was administered to the mothers of the IG at the T2 session post-treatment (*n* = 14). The raw scores and total score had good internal consistencies (treatment success: α = 0.83; treatment process: α = 0.81; total score: α = 0.89). Scale scores and the total score were compared with normative data.

#### 2.3.3. Parental Perceptions of Changes in Abdominal Pain

Mothers were asked about changes in their child’s abdominal pain symptoms (i.e., intensity, duration and frequency) in short, standardized interviews conducted by a psychologist at T1, T2 and T3. Answers were recorded on graded scales for a standardized recording of answers. Data from pre-intervention assessment (T1) referred to the last seven days and included pain location, intensity, frequency and duration. T1 data were then analyzed for a sample description. At post-intervention (T2) and follow-up (T3), parents were asked to rate the change of pain since the end of the intervention (“How did the abdominal pain change since the end of the intervention?”) with respect to the overall change of pain (very much improved; somewhat improved; not changed; somewhat worsened; very much worsened), perceived changes in pain intensity (intensity decreased; not changed; intensity increased), perceived changes in pain frequency (frequency decreased; not changed; frequency increased), and perceived changes in duration of pain (duration decreased; not changed; duration increased). These T2 and T3 data were analyzed descriptively and presented in the results section.

#### 2.3.4. Parent-Reported Outcome Measures

The following parent-reported outcome measures were assessed at all three points of measurement (T1–T3).

(1) Gastrointestinal Symptoms: Mothers were asked to report on their child’s GI symptoms in accordance with the current Rome-III classification (“My child was suffering during the last month from…”; six items: abdominal pain, diarrhea, constipation, nausea, vomiting, bloating; [33]). Items of this symptom list were answered on a five-point frequency scale (never, almost never, sometimes, often, almost always). We calculated a mean score as an indicator of GI symptom severity, which yielded an acceptable internal consistency (Cronbach’s alpha = 0.72).
(2) Parental pain-related behavior: A self-developed 24-item questionnaire was administered, covering different adaptive and maladaptive aspects of parental behavior and worries related to their child’s abdominal pain. Following the introductory sentence, “When my child has abdominal pain, …” mothers rated their reactions on a five-point Likert scale (never, seldom, sometimes, often, always). The inventory comprises the following six subscales: “protection” (five items, e.g., “let him/her stay home from school”; “allow things which are usually forbidden”; α = 0.77), “attention” (6 items, e.g., “I comfort my child”; α = 0.77), “medical help-seeking and home remedies” (4 items, e.g., ”I consult a doctor”; “I give him a hot water bottle”; α = 0.72), “support child’s coping” (3 items, e.g., “I support my child to distract on his own”; “I support my child to relax on his own”; α = 0.86) , and “worries” (6 items, e.g., “I am worried”; “I am concerned about my child’s future”; α = 0.83). Compilation of the items was based on literature of parent behavior [38,39] and pre-tested in another pilot-study [40]. Psychometric properties were sufficient with internal consistencies >0.70 for all scales and sufficient selectivity for all items (part-whole-correlations >0.30). As hypothesized, the scale “protection” showed significant positive correlations to attention (*r*_T1_ = 0.406, *p* = 0.029), medical help-seeking (*r*_T1_ = 0.404, *p* = 0.030) and worries (*r*_T1_ = 0.436, *p* = 0.018). The negative correlation to the scale “support child’s coping” was not statistically significant (*r*_T1_ = −0.189, *p* = 0.327).
(3) Parental pain-related self-efficacy: We adapted a validated, illness-specific self-efficacy scale for parents with children suffering from atopic dermatitis or asthma [41,42] to measure pain-specific self-efficacy expectations. The pain-specific self-efficacy scale was preceded by a short introduction (“Dealing with abdominal pain challenges you. We would like to know how confident you are about dealing with those challenges.”). The nine-item scale covers self-efficacy expectations with respect to preventive actions (two items; e.g., “My child spontaneously wants to do something that could worsen the pain. I am able to refuse his/her wish.”), being consequent (two items; e.g., “My child is in pain in the morning and doesn’t want to go to school. I am able to encourage my child to go to school.”), as well as intervening actions when the child is in acute pain (Five items; e.g., “My child is in hard pain. I am able to distract my child.”). The items were answered on a six-point Likert scale (“very unsure”–“very sure”). Internal consistency of the total score for parental pain-specific self-efficacy was acceptable (Cronbach’s alpha = 0.72).
(4) General self-efficacy: Self-efficacy was measured by the validated and broadly used General Self-Efficacy Scale (GSES; [43]). Schwarzer and Jerusalem [44] report internal consistencies from 0.82–0.93 and a 2-year retest-reliability of 0.47 (men) and 0.63 (women). The GSES total score is positively associated with self-esteem and optimism, and negatively correlated with anxiety and depression [43]. The GSES covers 10 items, enquiring about the subjective evaluation of one’s ability to cope with new or difficult demands in life (e.g., “When there are obstacles, I find my way to assert myself”; “I can find a solution for every problem”). Answer format was four-point (“not true”, “hardly true”, “somewhat true”, “exactly true”). Higher raw scores are associated with higher perceived self-efficacy. Internal consistency in our sample was excellent (Cronbach’s alpha = 0.92).
(5) Parental quality of life: We used the “Ulm Quality of Life Inventory for Parents of Chronically Ill Children” (ULQUIE) [45] to assess mothers’ self-reported quality of life. The questionnaire was developed for parents of chronically ill children and has been shown to discriminate between clinical and non-clinical groups, as well as different stages of illness [45]. The authors had reported acceptable to good internal consistency for the total score in the validation study on 244 parents of chronically ill children [45]. The 29 items have a five-point answer format (never, seldom, sometimes, often, always) and refer to the last seven days. The wording was adapted to our study and “chronic illness” was replaced by “abdominal pain”. We used the total score as a global measure for mothers quality of life, which yielded excellent internal consistency in our sample (Cronbach’s α = 0.91).

### 2.4. Data Analysis

Statistical analyses were carried out using SPSS Statistics (Version 22). All scale scores were transformed to the range of 0–100 to enhance comparability of measures within the study. Effects for primary and secondary outcomes were analyzed by two-factorial analysis of variance for repeated measures, using group (IG × WLC) and the factor time (T1 × T2 × T3) as a repeated measurement factor. Hypotheses refer to a significant group × time interaction (i.e., an increase or decrease of measures in mothers of the IG compared to the WLC over the three measurement points). In case of significant overall effects, changes from T1–T2, T1–T3 and T2–T3 in the outcome measure will be analyzed by post hoc tests, applying Bonferroni correction of alpha-level. Main effects for group and time will be reported as well. In case the assumption of sphericity is violated (Mauchly-Test), the Greenhouse–Geisser correction of degrees of freedom and significance level will be applied [46]. In an exploratory fashion, changes in child pain will be correlated with changes in maternal outcomes. For this, results with at least a statistical trend (*p* < 0.100) will be reported. Change will be measured by the mean difference in the outcomes between T1–T2 and T1–T3.

As previously mentioned, one child dropped out from the IG due to a physical injury after the second of six sessions. No other adverse events occurred. For this one case, the mother did not participate in the parent session and the parent report of the post-intervention and their 3 month follow-up is consequently missing. We replaced the missing data of this case for T2 and T3 according to the last observation carried forward (LOCF) method for an intention to treat (ITT) analysis (Figure 1). Besides the ITT analysis, data were also analyzed by per protocol analysis (PPA). Besides this one dropout, we were able to report a full retention rate. There was no a priori power calculation for this study, as power calculation was performed for child’s self-reported abdominal pain, which was our main outcome of the trial [31]. For the current analysis on maternal effects, we performed a post hoc power calculation and defined a power of 1-β >0.80 as sufficient. For each effect, Cohen’s *d* was used as a measure for effect size. Cohen’s *d* represents the treatment effect in units of standard deviations (*SDs*), with interpretation of Cohen’s *d* = 0.20 as a low, *d* = 0.50 as a moderate and *d* = 0.80 as a high effect. Significance level was set at alpha = 0.05.

## 3. Results

### 3.1. Sample and Baseline Pain Characteristics

The 29 randomized children were between 6.6 and 11.9 years old (*M* = 9.59, *SD* = 1.54), and predominantly girls (*n* = 25; 86.2%). All 29 children were suffering from abdominal pain for three months or longer. For the parents in the IG, all mothers participated in the parent sessions and in two cases, the fathers participated as well. With respect to the data analysis, only the mothers of the IG and WLC participated in the assessment sessions T1–T3. There were no missing data, except for the one dropout, and therefore 14/15 randomized mothers in the IG participated in the parent session. A summary of demographic and baseline pain characteristics can be found in Table 1.

### 3.2. Initial Analyses

All outcome measures were tested for a priori baseline differences between groups. There was a significantly higher baseline value in the IG only for the total score of quality of life (*t*(27) = 2.187, *p* = 0.038), which is included as a covariate in the repeated measures analysis for this outcome. Between mothers in the IG versus WLC, there were no baseline differences at T1 in the sum score of GI symptoms (*t*(27) = 1.111, *p* = 0.276), pain-related self-efficacy (*t*(27) = −1.725, *p* = 0.096) and general self-efficacy (*t*(27) = 0.297, *p* = 0.789). Mothers did also not differ on the baseline measures of pain-related behavior and worries (attention *t*(27) = −0.938, *p* = 0.357; protection *t*(27) = −1.678, *p* = 0.105; support child’s coping *t*(27) = −1.427, *p* = 0.165; medical help-seeking *t*(27) = −0.346, *p* = 0.732; worries *t*(27) = 0.683, *p* = 0.500).

#### Treatment Satisfaction

For the IG, the mothers’ evaluation of the intervention yielded high scores on the different scales of treatment success (*M* = 74.75, *SD* = 13.54, range 53.57–92.86) and treatment process (*M* = 96.05, *SD* = 4.49, range 87.50–100). The total score for treatment satisfaction was therefore high (*M* = 88.95, *SD* = 7.16, range 76.19–97.62). Taking the German cutoff values (percentile ranks: 0–10 clearly under average; 11–25 under average; 26–75 average; 76–90 over average; 91–100 clearly over average; [37]), treatment success was rated under average in 21.4% of participants; as average in 7.1%; as over average in 14.3% and as clearly over average in 57.1%. For the scale treatment process, 100% of ratings were over average. Taking the total score, 100% of ratings were categorized as clearly over average.

### 3.3. Primary Outcomes

#### 3.3.1. Maternal Perceptions of Changes in Pain

Analysis of the mother’s evaluation of changes in pain revealed highly significant differences between the groups, both for post-intervention T2 and for the follow-up assessment T3. As items were not assessed at T1, the dropout data could not be replaced in this analysis, resulting in 14 participants analyzed in the IG. For the overall change of pain at T2, 13/14 mothers in the IG reported that pain had very much improved (*n* = 1 reported somewhat improved), whereas in the WLC, 10/14 reported no change (*n* = 3 reported somewhat improved and *n* = 1 worsened; χ^2^(3) = 25.00, *p* < 0.001). Results at T3 were comparable. For the overall change at T3, 13/14 mothers in the IG reported that pain had very much improved (*n* = 1 reported somewhat improved), whereas in the WLC, 12/14 reported no change (*n* = 1 reported very much improved and *n* = 1 very much worsened; χ^2^(3) = 24.29, *p* < 0.001). A similar pattern was found when analyzing the data for pain intensity, frequency and duration (data not shown).

Table 2 summarizes the results of repeated measures ANOVA for the outcomes GI symptoms, pain-related self-efficacy, general self-efficacy and maternal quality of life. Results for maternal pain-related behavior and worries are summarized in Table 3.

#### 3.3.2. Gastrointestinal (GI) Symptoms

In line with our hypothesis, we found a significant decrease in the occurrence of child’s GI symptoms overtime, as perceived by the mothers, in the IG compared to the WLC (interaction *F*(2, 54) = 6.345, *p* = 0.003, *d* = 0.97). Of note, there was also an overall main time-effect (Table 2) and both groups showed a decrease from study inclusion to the T2 assessment (time effect *F*_1-2_(1, 27) = 12.871, *p*_1-2_ = 0.001, *d*_1-2_ = 1.38).

However, there was a significant decrease of GI symptoms in the IG during the follow-up time span between T2–T3 (interaction *F*_2-3_(1, 27) = 5.390, *p*_2-3_ = 0.028, *d*_2__-3_ = 0.89) and overall from T1 to T3 (interaction *F*_1-3_ (1, 27) = 13.128, *p*_1-3_ = 0.001, *d*_1-3_ = 1.39), with large effect sizes.

#### 3.3.3. Pain-Related Self-Efficacy

In line with our hypothesis, we found a significant and large-sized increase in maternal pain-related self-efficacy in dealing with child’s abdominal pain in the IG over time (group × time interaction *F*(1.58, 42.78) = 5.660, *p* = 0.011, *d* = 0.92). Post hoc analyses for the interaction term revealed significant changes with large effect sizes from T1 to T2 and overall from T1 to T3 (Table 2). While from T2 to T3 the scores further increased in the IG, the interaction term was not significant (*F*_2-3_(1, 27) = 1.101, *p*_2-3_ = 0.303, *d*_2-3_ = 0.40).

#### 3.3.4. Maternal Pain-Related Behavior

We found significant, large treatment effects (*d* = 0.92–*d* = 1.03) consistent with all hypotheses for attention, medical help-seeking, support child’s coping and worries. Post hoc analyses revealed that all changes were significant from T1 to T2 as well as overall from T1 to T3, with large effect sizes from *d* = 0.93–*d* = 1.33. Detailed results for the main effects (time, group) and treatment effect (group × time interaction, including post-hoc analyses for interaction) are summarized in Table 3. For the follow-up time span, the changes in the IG could be maintained and significant group differences with large effect sizes emerged, consistent with the direction of hypotheses (*d*_2-3_ = 2.10 for significant less attention in the IG, *d*_2-3_ = 1.22 for less medical help-seeking and home remedies, *d*_2-3_ = 0.94 for less worries and *d*_2-3_ = 0.80 for more support of child’s coping).

The scale “protection” was the only one for which results were not consistent with hypotheses, as the interaction term was not significant (*F*(2, 54) = 0.775, *p* = 0.466, *d* = 0.34). On a descriptive level, scores were reduced in the IG by on average 9.67 points, however, there were also large-sized differences on group level overall and at all time points (*F*_1-2_(1, 27) = 6.437, *p*_1-2_ = 0.017, *d*_1-2_ = 0.98; *F*_2-3_(1, 27) = 12.494, *p*_2-3_ = 0.001, *d*_2-3_ = 1.37); *F*_1-3_ (1, 27) = 8.331, *p*_1-3_ = 0.008, *d*_1-3_ = 1.11).

### 3.4. Secondary Outcomes

#### 3.4.1. General Self-Efficacy

Analysis of variance revealed a significant increase of general self-efficacy in both groups over time (main effect time *F*(2, 52) = 3.747, *p* = 0.030, *d* = 0.76). Scores increased from T1 to T3 on average by 5.78 (IG) respectively 4.36 (WLC) points. Changes in time were significant from T2 to T3 (*F*_2-3_(1, 26) = 5.078, *p*_2-3_ = 0.033, *d*_2-3_ = 0.88) and T1–T3 (*F*_1-3_(1, 26) = 5.438, *p*_1-3_ = 0.028, *d*_1-3_ = 0.92). There was no significant main effect for group (*F*(1, 26) = 0.146, *p* = 0.705), nor group × time interaction (*F*(2, 52) = 0.201, *p* = 0.818; Table 2).

#### 3.4.2. Maternal Quality of Life

Analysis of covariance controlling for the baseline T1 value did not show significant effects for time (*F*(1, 26) = 1.228, *p* = 0.278), group (*F*(1, 26) = 0.021, *p* = 0.886) or for group × time interaction (*F*(1, 26) = 0.497, *p* = 0.478). However, statistical power was insufficient, ranging from 1-β = 0.05–0.37 for the ULQUIE analyses (Table 2).

All presented results were derived from an ITT analysis. Result patterns of the PPA were similar, with increased effect sizes.

In an exploratory manner, we analyzed whether changes in the child’s pain corresponded with changes in maternal outcomes. Only results with *p* < 0.100 were reported. For the child’s pain, we used both the GI symptom score as perceived by the mother, as well as the child’s self-reported pain as a mean score covering pain intensity, frequency and duration per day. Data for the pain diary were derived from a previous publication [31] and are based on a 14 day pain diary. Bivariate correlations within the IG showed that for changes between T1 and T2, a pronounced decrease of self-reported abdominal pain was positively correlated with a pronounced decrease of maternal attention (*r* = 0.519, *p* = 0.047). A pronounced decrease of mother-reported GI symptoms corresponded with a higher increase in the support of child’s coping (*r* = −0.509, *p* = 0.052) and higher decrease in help-seeking (*r* = 0.464, *p* = 0.082), although not statistically significant. For the overall change between T1 and T3, a pronounced decrease in GI symptoms was significantly correlated with a pronounced decrease in help-seeking (*r* = 0.596, *p* = 0.088).

## 4. Discussion

While the efficacy of CBT on a child’s pain has been confirmed in several studies [17,18,20,47,48], only a few studies have reported on changes in parent outcomes [23,24,25]. This paper aimed to analyze whether any effects on the mothers of children suffering from FAP were noticeable, after participation in a group CBT focusing on their child’s self-management. We observed significant, large-sized effects, even though only one parent session was included. We first analyzed changes in the mother’s perception of her child’s symptom load. Mothers in the IG reported a clear improvement of abdominal pain and GI symptoms, which is in line with results for proxy reported pain in other treatment trials for FAP [23,24,49,50] and also corresponds to previously reported positive effects reported by the children [31].

We also assessed a broad range of pain-related maternal behaviors. According to the cognitive-behavioral pain model, hypotheses differentially referred to an increase of adaptive behavior, defined as supporting the child’s coping skills (i.e., distraction and relaxation), and a decrease of maladaptive behavior, defined as attention to pain, protective behavior and medical help-seeking. With the exception of protective reactions, all treatment effects on maternal behavior were significant, with large effect sizes and even maintained over the follow-up span. These results suggest that a broad range of maternal behavior can be influenced by even a short intervention. Palermo et al. [25] also reported similar significant, medium sized effects on parent’s encouragement of their child’s activities. In addition, in an uncontrolled study, Frerker et al. [51] observed long-term reductions in solicitous behavior and short-term increases in distraction after treatment. Contrary to hypotheses and other trials [23,24,25], no significant decrease of protective reactions in the IG compared to the WLC was observed. A floor effect might explain this result, since despite the fact that scores halved from pre-intervention to follow-up in the IG, the IG displayed very low scores already at pre-treatment, impeding further improvement.

Consistent with our hypothesis, the mothers in the IG also reported significantly less worries concerning their child’s abdominal pain at follow-up, which might imply that psychoeducation was successful (“pain is real, but not dangerous”). These results are promising, as there is evidence that the parent’s conceptual pain model is critical for their child’s prognosis [52]. Other CBT trials also report effects on parental cognitive-affective variables, like the reduction of perceived threats [24] and reduction of self-blame [25]. Participation in the training program also led to significant improvements in the mother’s pain-related self-efficacy, which is in line with research on other chronic diseases [41,42]. Thus far, no trial in childhood FAP research has included parent’s pain-specific self-efficacy as an outcome measure. As perceived self-efficacy constitutes an important variable in the context of chronic illness, explaining and predicting the implementation of treatment [28,42] and behavior change [27], further investigations of the parent`s pain-specific self-efficacy might give additional insight into other mechanisms of change.

Taking recent systematic reviews into account [21,22,53], evidence for treatment efficacy on parent and child outcomes, may also depend on the degree to which the parents are involved. Recent studies have reported that parental involvement varies between 25%–50% of total therapy time [53] and also varies with respect to the setting. In our intervention, parental involvement was comparably low, with a 6:1 ratio of child to parent therapy time (14% of total therapy time)—despite the young age of the children in our sample. Of note, trials can differ greatly with respect to the age range of participants. While we focused on younger children aged 6–12 years, others included a broader age range (e.g., aged 7–17 years [23] or only older children and adolescents aged 11–17 years [25]). However, the specific contents of these parent interventions are quite similar, covering, for instance, psychoeducation on FAP, the cognitive-behavioral model and discussions of parental behavior [23,49]. Since all other interventions have included more comprehensive parent sessions [22,53], the large-sized effects on parent outcomes are especially of interest. Perhaps parent involvement in FAP treatment does not need to be very extensive, as our results indicate that the approach of one accompanying parent session is still very effective.

In accordance with integrative views on childhood chronic pain [8,54], the effects of our intervention effect the child as well as the parent—which probably interact with one another in a self-reinforcing circle of positive effects. The improvement of maternal behavior might positively influence changes on the child level, while the reduction of child’s pain and impairment may be a motor for change in the mother’s behavior and worries. Levy et al. [55] found that changes in a parent’s threat perception significantly mediates changes in the child’s pain. Although these conclusions are limited due to the small sample size, exploratory correlation analyses provided promising first results on the mutual benefits of a child’s pain and maternal behavior. Future trials should systematically analyze both the child and parent effects of CBT interventions and mediational pathways, in order to gain further insight into possible reciprocal effects of treatment.

One further conclusion of this trial is that while we observed large-sized treatment effects on almost all abdominal pain-related measures, the constructs conceptualized on an unspecific, general level were not affected by treatment.

With respect to general self-efficacy, we did not observe an intervention effect. To put this in context with the level of self-efficacy, we compared the score of our total sample (*n* = 29) at baseline (*M* = 30.90, *SD* = 4.38; range 19–40) with the score of the normative sample derived from the validation study [56]. Paired *t*-test revealed that our sample reported even significantly higher general self-efficacy than the normative population-based score for adult women (*M* = 28.8, *SD* = 4.9; *p* = 0.015). Based on these results, the non-significant effects on general self-efficacy could be attributed to a ceiling effect. We can only speculate that the comparatively high level of self-efficacy might have catalyzed the significant changes found in pain-specific measures of self-efficacy and behavior in the IG. However, the potentially positive effects of general self-efficacy pointed out by other studies [57] needs to be analyzed in larger samples. In general, the different treatment responses of pain-specific versus general self-efficacy, as found in this study, are in line with the current literature. Since for chronic conditions, disease-specific measures are more sensitive to change [58,59] and accordingly, treatment effects for parents in the context of other pediatric chronic conditions have also been reported [60].

We also did not observe significant changes in the mother’s quality of life in our sample. This might be attributable to the fact that treatment content of the parent session was focused on pain-specific issues like the disease model and the child’s coping with pain, and therefore changes in the parent’s general well-being were not primarily focused on. One other reason might also be that for the mother’s quality of life, we already observed high levels at baseline. Exploratory comparisons to other ULQUIE data show that our sample, with *M* = 72.09 and *SD* = 10.72 for the total score in both groups at baseline, not only showed significantly higher scores than reported for parents of children with other chronic pediatric conditions [61,62], but also that values did not significantly differ from a healthy control sample (*M* = 74.0, *SD* = 28.6, *p* = 0.345; [61]). These data might suggest that parents in our trial were not severely strained to begin with. This is in line with results from a study on parental quality of life in a sample of parents with children suffering from inflammatory bowel disease who also reported higher quality of life scores in most of the domains [63]. On the other side, this observation contradicts other evidence on increased maternal psychosocial strain in FAP samples (e.g., [14,15,16]). It can be assumed that the relatively high scores on maternal quality of life in our study are attributable to the fact that recruitment for this trial took place in the general population, not in a clinical setting. To the best of our knowledge, there are few data on the quality of life of parents with children with FAP; therefore further studies with more representative samples are warranted.

This trial also has to be reviewed in the context of methodological strengths and limitations. We included various dimensions of parental experience and behavior as outcome measures and we were consequently able to draw differentiated conclusions, not only regarding maladaptive maternal reactions, but also regarding adaptive reactions and pain-specific self-efficacy. While the measure for parent behavior was a self-developed questionnaire based on clinical experience and literature, psychometric properties were satisfactory. Assessment of changes in child pain was conducted by an interview, which was standardized, but did not include established measures for pain assessment like a Numeral Rating Scale. Of note, the data on changes in pain are the mother’s subjective perception of her child’s pain level and not meant to be a validated proxy marker for the child’s pain experience. However, maternal perceptions of child’s pain are considered as important pathways for change and pain-related outcomes like healthcare seeking [23,64]. Additionally, this RCT was conducted according to CONSORT guidelines. The retention rate over the follow-up time span was excellent (96.6%) and there were no missing data on the questionnaires. The intervention was standardized and is available as manualized treatment program [29]. However, one should mention the comparably low sample size, with <20 individuals in each treatment arm, which can be traced back to the calculation of the sample size for the primary study outcome [31]; however, statistical power was sufficient for the main analyses in this paper (1-β > 0.80). As a common problem in pediatric pain research, only mothers participated in the assessment sessions and therefore we unfortunately do not have data of the fathers. However, Frerker et al. [51] did not find different changes in parental behavior after treatment in mothers versus fathers. Nonetheless, RCTs including a sufficient number of fathers are warranted to enable conclusions with respect to gender-related treatment effects. In addition, interpretation and generalizability of results may be constrained due to the selectivity of the non-treatment-seeking sample with a high rate of decliners for further examination and single-site conduction of this trial. Replication of results, including a multi-site approach with a one-year follow-up and active control group, is currently ongoing [65].

The evaluations of our CBT program gave not only promising evidence for its efficacy, but it also identified possible decisive catalysts for improvement, which should be targeted in further clinical practice. Of note, we cannot imply the degree of parent involvement which is needed, or whether we need a child or a parent focus. Nonetheless, child’s self-management, together with parent interventions such as psychoeducation on FAP, and taking parental concerns seriously, such as discussing everyday demands and working on adaptive alternatives, seem to constitute promising strategies for improvement of the psychosocial situation of both children and parents. Identification of the mechanisms at work in the circle of positive effects can deliver valuable implications for management of FAP in pediatric practice and also amend integrative parent-child approaches in childhood FAP [8].

## 5. Conclusions

Functional abdominal pain in children is a common and impairing condition, affecting not only the children themselves but also the parents. Taking into account the growing evidence on parent-specific changes in pediatric pain management, as well as the positive effects of our CBT program on the child level that have recently been published, this study aimed to analyze whether the mothers randomized to the CBT IG for their child’s FAP, may benefit from participation in the program as well. Despite a strong focus on child’s self-management, large-sized effects on mother’s behavior, worries and self-efficacy in dealing with child’s pain were observed in the IG, whereas for the mothers in the waitlist control group, no changes were observed. Of note, despite these pain-specific effects, the mother’s perception of their self-efficacy in everyday life and their quality of life was not significantly affected.

These results add to the current knowledge on treatment changes experienced by mothers of children suffering from FAP. The fact that, in our sample, the mothers strongly benefited from the training program, together with previously reported child benefits, implies that the circle many families find themselves in can be broken with these interventions. Extending this line of research and taking a broader family perspective [8,9], a thorough investigation of treatment effects on the family level are also warranted. Therefore, additional studies are needed to gain a better understanding of mechanisms of FAP treatment.

## Figures and Tables

**Table 1:** Demographic data and baseline pain characteristics of the sample (*n* = 29).

Variables	IG (*n* = 15)	WLC (*n* = 14)	*p*-Value
**Single parent**			0.280
yes	14	11	
no	1	3	
**Educational level mother**			0.362
≤10 years	5	7	
>10 years	10	7	
**Educational level father**			0.909
≤10 years	4	4	
>10 years	11	10	
**Job situation mother**			
not employed	1	1	0.960
(self-) employed	14	13	
**Job situation father**			
not employed	0	0	NA
(self-) employed	15	14	
**Number of siblings**			
*M* (*SD*)	1.79 (1.67)	1.50 (0.76)	0.566
range	0–7	1–3	
**Psychological distress of the child ^1^**			
**Total problem score**			
Normal	8	11	0.415
Borderline	5	2	
Abnormal	1	1	
**Abdominal pain intensity (last 7 days)**			
*M (SD)*	3.00 (1.00)	2.64 (1.28)	0.407
range	1–4	1–5	
Abdominal pain frequency *			
*M*	9.13	11.61	0.396
*SD*	(4.91)	(9.55)	
range	2–20	3–30	
Abdominal pain duration **			
*M (SD)*	57.13 (34.41)	61.79 (73.00)	0.826
range	15–120	10–300	

All variables were collected from the mother’s report. All frequencies are depicted for the number of cases *n*; for metric variables, mean (*M*), standard deviation (*SD*) and range are depicted. *p*-value refers to the significance value according to χ^2^/*t*-test when *p* < 0.05. NA, not applicable (as there were no cases of unemployed fathers, group comparisons were not conducted); IG, intervention group; WLC, wait list control group. ^1^ data on psychological distress (*n* = 14 in the IG due to missing item) are based on German normative values [35] and are published for the total sample in [30]; * number of days last month; ** minutes on average.

**Table 2:** Outcome measures (means, *SDs*) with results of repeated measures ANOVA (main effects for group, time, and group × time interaction).

	IG *n* = 15		WLC *n* = 14		Main Effect Time	Main Effect Group	Group × Time Interaction			
							*p* ^a^/*d* ^a^	Post Hoc Test ^b^		
	*M*	SD	*M*	SD	*p/d*	*p/d*		*F*	*p*	*d*
**Gastrointestinal Symptoms**										
Baseline	32.22	11.72	26.49	15.90	<0.001/1.36	0.492/0.27	0.003/0.97	1.722 (1–2)	0.201 (1–2)	0.51 (1–2)
Post	18.61	11.12	20.24	12.43				5.390 (2–3)	0.028 (2–3)	0.89 (2–3)
Follow-up	11.67	9.61	23.81	16.24				13.128 (1–3)	0.001 (1–3)	1.39 (1–3)
**Pain-related self-efficacy**										
Baseline	65.33	12.79	72.22	7.98	0.059/0.70	0.730/0.13	0.011/0.92	5.483 (1–2)	0.027 (1–2)	0.90 (1–2)
Post	73.63	11.93	70.00	9.58				1.101 (2–3)	0.303 (2–3)	0.40 (2–3)
Follow-up	77.63	13.92	70.79	10.38				7.496 (1–3)	0.011 (1–3)	1.05 (1–3)
**General self-efficacy**										
Baseline	70.44	15.78	69.74	13.84	0.030/0.76	0.705/0.16	0.818/0.18	NA	-	-
Post	73.33	12.60	70.25	13.43				-	-	-
Follow-up	76.22	15.47	74.10	16.79				-	-	-
**Quality of life**										
Baseline	76.03	6.73	67.86	12.70	0.278/0.43	0.886/0.06	0.487/0.28	-	-	-
Post	71.55	11.40	65.70	10.96				-	-	-
Follow-up	77.87	11.25	72.29	14.78				-	-	-

*p*, significance level; *d*, effect size. All scores were transformed to a range of 0–100. ^a^ for group × time interaction; when violated sphericity, df and *p*-value correction (Greenhouse–Geisser); ^b^ test of within-subject contrasts for interaction, with 1 = T1; 2 = T2; 3 = T3. NA, post-hoc analyses not applicable due to non-significant group × time interaction.

**Table 3:** Mothers’ pain-related behavior and worries (means, *SDs*) with results of repeated measures ANOVA (main effects for group, time, and group × time interaction).

	IG *n* = 15		WLC *n* = 14		Main Effect Time	Main Effect Group	Group × Time Interaction			
							*p* ^a^/*d* ^a^	Post Hoc Test ^b^		
	*M*	SD	*M*	SD	*p/d*	*p/d*		*F*	*p*	*d*
**Attention**										
Baseline	51.39	19.71	57.14	12.17	0.001/1.04	<0.001/1.59	0.004/0.95	9.746 (1–2)	0.004 (1–2)	1.20 (1–2)
Post	34.72	12.27	58.93	16.00				0.044 (2–3)	0.835 (2–3)	0.09 (2–3)
Follow-up	31.39	13.81	54.46	12.71				7.320 (1–3)	0.012 (1–3)	1.04 (1–3)
**Protection**										
Baseline	17.67	17.61	28.93	18.52	0.109/0.59	0.0004/1.21	0.466/0.34	NA	–	–
Post	12.33	15.34	29.29	17.41				–	–	–
Follow-up	8.00	9.60	26.07	16.66				–	–	–
**Support coping**										
Baseline	51.67	19.21	60.12	11.40	<0.001/1.17	0.0342/0.37	0.002/1.03	8.147 (1–2)	0.008 (1–2)	1.10 (1–2)
Post	69.44	17.16	59.52	15.28				0.276 (2–3)	0.603 (2–3)	0.20 (2–3)
Follow-up	75.56	17.10	62.50	17.30				11.994 (1–3)	0.002 (1–3)	1.33 (1–3)
**Help-seeking**										
Baseline	28.75	15.99	30.80	15.97	<0.001/1.46	0.032/0.87	0.005/0.92	5.891 (1–2)	0.022 (1–2)	0.93 (1–2)
Post	12.08	10.94	25.00	13.31				1.100 (2–3)	0.304 (2–3)	0.40 (2–3)
Follow-up	11.67	11.54	28.57	16.39				8.108 (1–3)	0.008 (1–3)	1.10 (1–3)
**Worries**										
Baseline	41.11	19.79	36.01	20.39	<0.001/1.69	0.222/0.48	0.002/1.03	7.133 (1–2)	0.013 (1–2)	1.03 (1–2)
Post	21.11	17.21	32.44	16.19				0.444 (2–3)	0.511 (2–3)	0.26 (2–3)
Follow-up	14.72	13.90	28.87	13.02				10.120 (1–3)	0.004 (1–3)	1.23 (1–3)

*p*, significance level; *d*, effect size. All scores were transformed to a range of 0–100. “support coping” refers to the scale “support child’s coping”; “help-seeking” refers to the scale “medical help-seeking and home remedies”; ^a^ for group × time interaction; when violated sphericity, df and *p*-value correction (Greenhouse–Geisser); ^b^ test of within-subject contrasts for interaction, with 1 = T1; 2 = T2; 3 = T3. NA, post-hoc analyses not applicable due to non-significant group × time interaction.

## Supplementary Materials

The following is available online at www.mdpi.com/2227-9067/4/2/13/s1, Table S1: CONSORT Checklist.
